# Recombinant Amelogenin as a Potential Alternative to Enamel Matrix Derivatives in Periodontal Regeneration: A Scoping Review of Its Biological Activity, Synthesis and Delivery Systems

**DOI:** 10.1002/cre2.70325

**Published:** 2026-03-06

**Authors:** Jie Dan Denny Luo, Jennifer Mardini, Raymond Lee, Fariba Dehghani, Aaron Schindeler

**Affiliations:** ^1^ School of Chemical and Biomolecular Engineering The University of Sydney Sydney Australia; ^2^ Faculty of Medicine and Health, Sydney Dental School University of Sydney Sydney Australia; ^3^ Bioengineering and Molecular Medicine Laboratory The Children's Hospital at Westmead and Westmead Institute for Medical Research Sydney Australia

**Keywords:** in vitro, in vivo models, periodontal regeneration, recombinant amelogenin

## Abstract

**Objective:**

The objective of this review is to evaluate the existing literature on the regenerative potential of recombinant amelogenin, with a particular emphasis on the biological activity, synthesis, and delivery systems relevant to periodontal regeneration.

**Materials and Methods:**

A comprehensive literature search was conducted across PubMed, Ovid MEDLINE, Scopus and Web of Science to identify studies on recombinant amelogenin for periodontal regeneration. Inclusion criteria encompassed in vitro and animal model studies reporting on cellular growth, regeneration markers, tissue repair and clinical improvements. Data extraction focused on study design, protein source, delivery system, experimental model, and reported biological outcomes. The review was conducted in accordance with the PRISMA‐ScR guidelines and supplemented by a narrative synthesis of the preclinical literature.

**Results:**

The literature search included 23 studies comprising predominantly preclinical cell culture and in vivo mouse studies. Recombinant amelogenin was evaluated across diverse experimental systems and delivery methods, most frequently by direct dissolution in culture media, with fewer studies using injectable hydrogels or scaffold‑based carriers. Although most investigations reported biological activity attributable to recombinant amelogenin, the outcome measures, experimental models, and formulation strategies varied substantially, limiting direct comparison across studies.

**Conclusion:**

Recombinant amelogenin shows consistent preclinical activity supporting its biological plausibility as a regenerative agent. However, evidence remains largely preclinical and heterogeneous. Future studies should employ standardised models, optimised delivery systems, and direct comparisons with established therapies to clarify its translational potential in periodontal regeneration.

## 1 Introduction

### 1.1 Rationale

Periodontitis is a prevalent chronic inflammatory disease that leads to the progressive destruction of the periodontal ligament, alveolar bone, and supporting connective tissue, ultimately resulting in tooth loss (Tonetti et al. 2018). Traditional regenerative therapies, such as guided tissue regeneration and bone grafting, have demonstrated variable success, often constrained by biological limitations, operator‐dependent techniques, and patient‐specific factors (Nibali et al. 2020). Biologic agents, particularly enamel matrix derivatives (EMDs), have emerged as promising adjuncts in periodontal regeneration by promoting the regeneration of lost periodontal tissues where human histological evidence supports their ability to induce true periodontal regeneration, including the formation of new cementum, periodontal ligament, alveolar bone, and the insertion of Sharpey's fibres across the periodontal apparatus (Miron et al. 2016). The principal protein in EMDs, amelogenin, plays a key role in the formation and organisation of dental hard tissues and has shown potential in promoting periodontal regeneration. Commercially available EMDs, such as Emdogain (Straumann), have been extensively studied over the past 25 years, with clinically proven effects confirmed by large multicentre trials, human histological evidence, and meta‐analysis of randomised clinical trials (Esposito et al. 2009; Sculean et al. 1999; Silvestri et al. 2003). Emdogain consists of EMDs suspended in a ready‐to‐use gel containing a delivery solution, propylene glycol alginate (PGA). Notably, the delivery system (PGA) exhibits significant antimicrobial effects against periodontal pathogens, although the original studies attributed these effects to Emdogain itself (Esposito et al. 2009; Sculean et al. 2001; Spahr et al. 2002).

Recombinant expression systems have enabled the production of human amelogenin with improved consistency and scalability compared to traditional EMDs (Taylor et al. 2006). This approach avoids the use of animal‐derived proteins, reducing ethical concerns and the risk of xenogeneic immune responses (Haze et al. 2009; Taylor et al. 2006). Taylor et al. (2006) demonstrated the successful production of biologically active recombinant human amelogenin using a baculovirus system in *Spodoptera frugiperda* (Sf9) insect cells, achieving yields of up to 10 mg/L. The recombinant protein was validated by SDS‐PAGE, Western blotting, ESI‐TOF mass spectrometry, peptide mapping, and tandem MS, confirming its purity and functional integrity (Haze et al. 2009). This method enhances the reproducibility and safety profile of amelogenin‐based products and supports their application in periodontal regeneration. Nevertheless, xeno‐derived EMDs rather than recombinant amelogenin remain the standard of care in periodontal clinical practice.

This review evaluates current evidence on the use of recombinant amelogenin in periodontal regeneration. While it offers potential advantages in consistency, safety and scalability over tissue‐purified EMDs, its clinical efficacy depends heavily on the formulation and delivery method. Variability in release kinetics, protein stability and bioactivity across delivery systems complicates the interpretation of the literature. Although several preclinical studies have explored recombinant amelogenin‐based therapies, findings remain fragmented, with few direct comparisons of delivery vehicles or integration strategies within composite regenerative biomaterials.

### 1.2 Objectives

The objective of this review was to evaluate and map the existing preclinical and early translational literature on the regenerative potential of recombinant amelogenin in periodontal therapy. Specifically, this review aimed to summarise the evidence on the biological effects of recombinant amelogenin in both in vitro and in vivo models of periodontal regeneration, as well as examine the protein synthesis or engineering involved, and characteristics of delivery systems employed and their influence on regenerative outcomes.

## 2 Methods

### 2.1 Registration and Protocol

A scoping review approach was used to map biological effects, delivery strategies, and periodontal regenerative outcomes, while documenting methodological quality and translational gaps as recombinant amelogenin research remains largely preclinical and mechanistic. This scoping review was therefore conducted in accordance with the PRISMA Extension for Scoping Reviews (PRISMA‐ScR) (Tricco et al. 2018). The review protocol was registered on OSF (Open Science Framework), and the registration DOI is 10.17605/OSF.IO/D9VFT.

### 2.2 Eligibility Criteria

The inclusion criteria used in this literature review for studies selection were based on the PICOS method and were the following:

– (P) Population: studies involving any populations (animal models, human subjects, and in vitro cell‐based studies) involved in periodontal regenerative treatment or intervention
– (I) Intervention: studies investigating recombinant amelogenin or related recombinant proteins or biomaterials used for periodontal regeneration
– (C) Comparisons: studies comparing the effects of recombinant amelogenin or related treatments with alternative therapies for periodontal regeneration or other regenerative approaches, including non‐recombinant commercially available treatments (EMDs)
– (O) Outcome measures: studies reporting on the effectiveness of recombinant amelogenin in regenerating periodontal tissues, including in vitro measures of cellular growth and regeneration markers. In vivo outcomes include tissue repair, regeneration success, clinical improvements and histological findings
– (S) Types of studies: all studies were considered

Studies were excluded if they were published in a language other than English, did not provide access to the full‐text manuscript, or focused on the use of recombinant amelogenin for purposes other than the regeneration of periodontal tissues. Additionally, two studies were excluded as they investigated the production of amelogenin without testing its regenerative effects or were systematic or narrative review articles.

### 2.3 Information Sources

An electronic search into four databases: PubMed, Ovid MEDLINE, Scopus and Web of Science was performed to systematically identify the available literature. All articles published up until 8th January 2026, were considered.

### 2.4 Search Strategy

The search string comprised the combination of medical subject headings (MeSH) and free keywords. The linkage was conducted using the Boolean operator (AND, OR). The choice of keywords was intended to be broad to maximise the number of relevant studies considered. The keywords were:

– recombinant: “recombinant”[All Fields] OR “recombinants”[All Fields] OR “recombinate”[All Fields] OR “recombinated”[All Fields] OR “recombinates”[All Fields] OR “recombination, genetic”[MeSH Terms] OR (“recombination”[All Fields] AND “genetic”[All Fields]) OR “genetic recombination”[All Fields] OR “recombination”[All Fields] OR “recombinations”[All Fields] OR “recombinational”[All Fields] OR “recombinative”[All Fields] OR “recombine”[All Fields] OR “recombined”[All Fields] OR “recombineered”[All Fields] OR “recombineering”[All Fields] OR “recombines”[All Fields] OR “recombining”[All Fields]
– amelogenin: “amelogenin”[MeSH Terms] OR “amelogenin”[All Fields] OR “amelogenins”[All Fields]
– periodontal: “periodontal”[All Fields] OR “periodontally”[All Fields] OR “periodontically”[All Fields] OR “periodontics”[MeSH Terms] OR “periodontics”[All Fields] OR “periodontic”[All Fields] OR “periodontitis”[MeSH Terms] OR “periodontitis”[All Fields] OR “periodontitides”[All Fields]
– regeneration: “regenerability”[All Fields] OR “regenerable”[All Fields] OR “regenerant”[All Fields] OR “regenerants”[All Fields] OR “regenerate”[All Fields] OR “regenerated”[All Fields] OR “regenerates”[All Fields] OR “regenerating”[All Fields] OR “regeneration”[MeSH Terms] OR “regeneration”[All Fields] OR “regenerations”[All Fields]

The following search strategy was applied to PubMed, Ovid MEDLINE, Scopus and Web of Science:

(“recombinant”[All Fields] OR “recombinants”[All Fields] OR “recombinate”[All Fields] OR “recombinated”[All Fields] OR “recombinates”[All Fields] OR “recombination, genetic”[MeSH Terms] OR (“recombination”[All Fields] AND “genetic”[All Fields]) OR “genetic recombination”[All Fields] OR “recombination”[All Fields] OR “recombinations”[All Fields] OR “recombinational”[All Fields] OR “recombinative”[All Fields] OR “recombine”[All Fields] OR “recombined”[All Fields] OR “recombineered”[All Fields] OR “recombineering”[All Fields] OR “recombines”[All Fields] OR “recombining”[All Fields]) AND (“amelogenin”[MeSH Terms] OR “amelogenin”[All Fields] OR “amelogenins”[All Fields]) AND (“periodontal”[All Fields] OR “periodontally”[All Fields] OR “periodontically”[All Fields] OR “periodontics”[MeSH Terms] OR “periodontics”[All Fields] OR “periodontic”[All Fields] OR “periodontitis”[MeSH Terms] OR “periodontitis”[All Fields] OR “periodontitides”[All Fields]) AND (“regenerability”[All Fields] OR “regenerable”[All Fields] OR “regenerant”[All Fields] OR “regenerants”[All Fields] OR “regenerate”[All Fields] OR “regenerated”[All Fields] OR “regenerates”[All Fields] OR “regenerating”[All Fields] OR “regeneration”[MeSH Terms] OR “regeneration”[All Fields] OR “regenerations”[All Fields]).

### 2.5 Selection Process

Study selection followed a two‐step approach: (a) screening of titles and abstracts, and (b) full‐text analysis, with reasons for exclusion reported. The titles and abstracts of each research article in the initial search were independently screened by the primary reviewer (D.L.), while a second reviewer (J.M.) cross‐checked the selections to ensure consistency and reduce bias. Any conflicts or discrepancies between reviewers were addressed by a third reviewer (R.L.).

The full‐text manuscripts of the articles were catalogued using EndNote for reference management and assessed in accordance with the Eligibility Criteria outlined above. The articles were evaluated based on the following criteria:

– The study clearly describes the source, production or synthesis of recombinant amelogenin and its carrier for therapeutic, clinical or investigatory use.
– The study comprehensively discusses the delivery system used for recombinant amelogenin, focusing on how the carrier system influences the stability, release rate and bioactivity of recombinant amelogenin, as well as its biocompatibility.
– The study describes the in vitro tests used to evaluate cellular responses and biomolecular markers of regeneration associated with recombinant amelogenin, as well as any in vivo or clinical tests conducted in animals or humans to assess its therapeutic performance.

No automation tools were used in the initial screening process, aside from EndNote for managing references and organising the workflow.

### 2.6 Data Collection Process

The full‐text manuscripts of the included studies were catalogued chronologically. Data from these studies were independently extracted by the primary reviewer (D.L.) according to the Data Items listed below, while a second reviewer (J.M.) cross‐checked the extractions to ensure consistency and reduce bias. Any conflicts or discrepancies between reviewers were addressed by a third reviewer (R.L.). No automation tools were used.

### 2.7 Data Items

The data collected from the included studies were arranged in the following fields:

– Author (Year): Author(s) and year of publication
– Study context and model: Study type (in vitro/in vivo), cell type or animal model and study aim
– Recombinant amelogenin characteristics: Species origin, expression or synthesis system, variants or modifications and characterisation methods (if reported)
– Delivery system/formulation: Mode of application, carrier material and formulation characteristics
– Methodological notes: Key design features, reporting characteristics and study limitations
– Outcome domains: In vitro biological and biomolecular responses and in vivo tissue regeneration outcomes
– Critical appraisal: Summary of authors' conclusions

### 2.8 Study Risk of Bias Assessment

The risk of bias in the included studies was assessed using two validated tools appropriate to the study type. For in vitro studies, the QUIN Tool (Quality Assessment Tool for In Vitro Studies) was used to evaluate methodological quality and potential sources of bias (Sheth et al. 2024). Based on the total score, studies were categorised into three risk levels: low risk (> 70%), medium risk (50%–70%), and high risk (< 50%) (Sheth et al. 2024). For animal studies, SYRCLE's Risk of Bias (RoB) Tool was applied, which is specifically designed for preclinical studies involving animals (Hooijmans et al. 2014). This tool evaluates risk across six domains: selection, performance, detection, attrition, reporting and other. Each domain is rated as ‘yes’ (low risk), ‘no’ (high risk), or ‘unclear’ (insufficient information) (Hooijmans et al. 2014). A ‘yes’ judgement indicates low risk of bias, ‘no’ indicates high risk of bias, and ‘unclear’ indicates insufficient details to assess the risk of bias (Hooijmans et al. 2014). The overall risk of bias was classified into three categories:

- **Low**: All domains rated as ‘low risk’ or only 1–2 domains rated as ‘unclear’, with no domains rated as ‘high risk’.
- **Unclear**: One or more domains rated as ‘unclear’, with no domains rated as ‘high risk’.
- **High**: One or more domains rated as ‘high risk’.

Each included study was independently assessed by the primary reviewer (D.L.). A second reviewer (J.M.) cross‐checked the assessments to ensure consistency and reduce the potential for subjective bias. Any disagreements were resolved through discussion, with a third reviewer (R.L.) acting as adjudicator where consensus could not be reached.

No automation tools were used during the risk of bias assessment.

### 2.9 Effect Measures

The primary approach for evaluating outcomes was qualitative or descriptive synthesis. The following outcome domains were defined, with relevant data extracted accordingly:

- In vitro biological responses and biomolecular markers: this domain captured cellular behaviours and molecular outcomes assessed following exposure to recombinant amelogenin, including cell proliferation, viability, migration, attachment and differentiation, as well as the expression of genes and proteins associated with periodontal and mineralised tissue regeneration. These included growth factors, extracellular matrix components, and markers related to osteogenic or cementogenic activity. All reported assays and evaluated time points were extracted to describe patterns in experimental focus and outcome reporting.
- In vivo tissue regeneration outcomes: this category encompassed histological, radiographic and clinical assessments of periodontal tissue regeneration in animal models. Data included measures of new bone formation, cementum and periodontal ligament regeneration, clinical attachment gain and defect fill.
- Protein synthesis and characterisation: this domain mapped how recombinant amelogenin was produced and characterised across studies, including expression systems, purification methods, post‐translational modifications (where reported) and protein variants. These data were extracted to identify trends and gaps in recombinant protein design relevant to translational development.
- Delivery systems and formulation strategies: This domain captured the types of carriers, scaffolds or formulation approaches used to deliver recombinant amelogenin, including direct application, hydrogels, injectable matrices and scaffold‐based systems. Information on formulation properties, application methods and intended release characteristics was extracted to describe how delivery strategies have been explored in relation to biological and regenerative outcomes.

### 2.10 Synthesis Methods

A quantitative meta‐analysis was not performed due to the exploratory scope and substantial heterogeneity of the available evidence, which rendered quantitative pooling inappropriate. Instead, findings were synthesised using descriptive and narrative mapping approaches to characterise how recombinant amelogenin has been investigated across different experimental contexts. To support transparent comparison across heterogeneous studies, results were first grouped according to experimental setting (in vitro vs. in vivo/preclinical models) and organised within each group under pre‐defined evidence domains, including biological and biomolecular responses, tissue‐level regenerative outcomes, protein synthesis and characterisation and delivery systems or formulation strategies. Within this framework, a semi‐quantitative effect‐direction (vote‐count) synthesis was performed for key outcome domains. For in vitro studies, these included cellular responses (proliferation, viability, migration, attachment and differentiation), biomolecular markers associated with osteogenic or cementogenic activity and mineralisation. For in vivo studies, outcomes focused on periodontal tissue regeneration, including new bone, cementum and/or periodontal ligament formation. For each study, outcomes were categorised according to the reported direction of effect relative to controls (positive, no difference/mixed or negative), and counts were tabulated by study type (in vitro or animal). This approach complements the narrative synthesis by illustrating consistency in directional findings without implying pooled effect estimates.

## 3 Results

### 3.1 Study Selection

**Figure 1:**
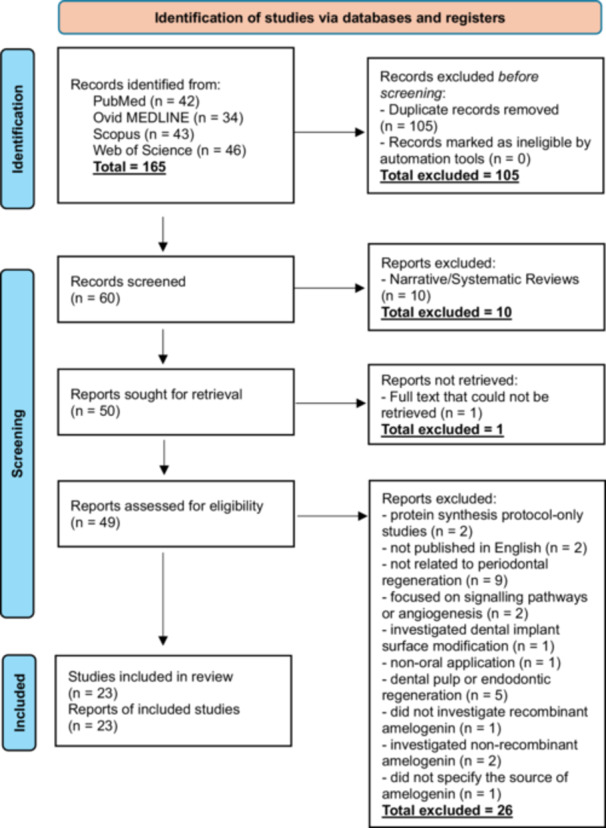
*PRISMA* flow diagram.

The electronic search in the databases of PubMed (*n* = 42), Ovid MEDLINE (*n* = 34), Scopus (*n* = 43), and Web of Science (*n* = 46) resulted in the identification of 162 potential titles and abstracts. After removal of the duplicates (*n* = 105), screening of the abstracts resulted in the selection of 60 studies for full‐text assessment of eligibility. A total of 37 studies were excluded after full‐text screening for the following reasons: narrative or systematic reviews (*n* = 10); protein synthesis protocol‐only studies (*n* = 2); full text could not be retrieved (*n* = 1); not published in English (*n* = 2); studies were not related to periodontal regeneration (*n* = 9), studies focused solely on signalling pathways or angiogenesis (*n* = 2); a study that investigated an unrelated topic around dental implant surface modification (*n* = 1); non‐oral application (*n* = 1); studies focused on dental pulp or endodontic regeneration (*n* = 5); one study did not investigate recombinant amelogenin (*n* = 1); studies that investigated non‐recombinant amelogenin (*n* = 2); and study did not specify the source of amelogenin (*n* = 1). A total of 23 studies were eligible and are included in the review (Figure 1 PRISMA flow diagram). Of these, 20 studies incorporated an in vitro experimental component, including 17 in vitro‐only studies and three studies that combined in vitro and animal experimentation. Six studies included an in vivo animal component, comprising three animal‐only studies and three preclinical studies with both in vitro and in vivo arms. No human clinical studies were identified.

### 3.2 Study Characteristics

Across the 23 included studies, recombinant amelogenin was investigated exclusively in in vitro systems and animal models, with no human clinical studies identified.

Study characteristics and key findings are summarised in Table 1, aligned with the predefined data items and effect measures described in Section Materials and Methods, 2. A critical appraisal of study design and reporting quality is included to contextualise findings within the overall evidence base.

**Table 1:** Critical analysis table for each included study (*n* = 23).

Author (year)	Study context and model	Recombinant amelogenin characteristics	Delivery system/formulation	Methodological notes	Outcome domains assessed	Critical appraisal
Zeichner‐David et al. (2006)	**Type:** In vitro experimental study **Cell type:** Periodontal ligament (PDL) cells derived from molars of 6‐week‐old Immortomice **Study aim:** To investigate the effects of recombinant mouse amelogenin (mrAMGN) and ameloblastin (mrAMBN) on PDL cell adhesion, proliferation, and differentiation **Controls:** Albumin, collagen, and media alone	**Species:** Mouse **Expression system/synthesis method:**	Directly dissolved in culture media	**Cell attachment and proliferation:** WST‐1 assay; tested mrAMGN up to 20 μg/mL, mrAMBN up to 30 μg/mL **Differentiation:** Cells cultured with ascorbic acid and β‐glycerophosphate; morphological analysis at Days 7, 14, 21, 28; osteogenic marker expression (BMP, OCN, BSP) assessed by RT‐PCR **Limitations:** In vitro only; protein stability and dosage effects not fully explored; no statistical comparison of combined vs. individual proteins; no comparison with commercial EMD	**In vitro biological responses and biomolecular markers:**	The study reveals that: mrAMGN and mrAMBN can induce growth‐factor‐like activity in PDL cells. They enhance attachment, stimulate proliferation, and induce differentiation suggesting roles in periodontal regeneration. mrAMBN may be more stable and favourable for prolonged culture as mrAMGN may be more transiently active.
					– **Cell attachment:** mrAMGN and mrAMBN increased attachment 13%–18% vs. control; no effect from albumin or collagen – **Cell proliferation:** mrAMGN (15 μg/mL) increased proliferation 9%; mrAMBN (≥ 4 μg/mL) up to 16% – **Cell differentiation:** Morphological changes included spindle‐/stellate‐shaped cells (Day 14), bundle formation (Day 21),clustering with mrAMBN or combined treatment (Day 28). mrAMGN alone induced cell death by Day 28. RT‐PCR showed upregulation of osteogenic markers, especially with mrAMBN or combined treatment	
		– **Amelogenin (mrAMGN):** Mouse amelogenin cDNA (M180) cloned into pET11a, expressed in *E. coli* BL21(DE3) with IPTG induction, purified via preparative and analytical C4 reverse‐phase HPLC. – **Ameloblastin (mrAMBN):** Mouse ameloblastin cDNA cloned into pMT/BiP/V5‐His B vector with C‐terminal His₆ tag; secreted protein under MT promoter.				
		**Variants:** Full‐length amelogenin (ME179); full‐length His‐tagged ameloblastin **Characterisation:** Confirmed by amino acid analysis, Edman sequencing, and MALDI‐TOF mass spectrometry				
Haze et al. (2009)	**Type:** Experimental animal study **Animal model:** 6 beagle dogs (13–16 months old, 9–10 kg) with experimentally induced periodontitis; degree III furcation defects created using wire ligature **Study aim:** To investigate the regenerative capacity of recombinant human amelogenin protein (rHAM) in periodontal defects **Control:** Propylene glycol alginate (PGA) carrier	**Species:** Human **Expression system/synthesis method:** Bac‐to‐Bac baculovirus system in Sf9 insect cells; human amelogenin cDNA (175‐aa isoform) cloned into pFastBac HTb; cells lysed in GuHCl buffer; protein purified via Ni–NTA affinity chromatography and FPLC; optional His‐tag removal with TEV protease; dialysed against 50 mM acetic acid and stored at −70°C **Variants:** Full‐length 175‐aa isoform **Characterisation:** Sequence verified, purified protein confirmed by standard protein purification techniques	rHAM (100 µg) dissolved in 0.1 mL PGA carrier and applied directly to defect site during periodontal flap surgery	**Experimental design:** Periodontitis induced in all quadrants; after 6 weeks, periodontal flap surgery performed with root debridement, EDTA root surface demineralisation, and rHAM application in one quadrant per dog; PGA alone applied to control quadrants **Sample collection:** Dogs euthanised at 2, 4, and 8 weeks; jaws dissected into blocks, fixed in paraformaldehyde, and processed for histology, immunohistochemistry, in situ hybridisation, and micro‐CT analysis **Limitations:** Small sample size; no power calculation; limited follow‐up (< 12 weeks) for tissue maturation; no comparison with commercial EMD products	**In vivo tissue regeneration outcomes:**	The study provides clear preclinical evidence for rHAM as a biological regenerative agent in periodontal defects showing tri‐tissue (cementum, periodontal ligament, and alveolar bone) regeneration in a dog model.
					– **Alveolar bone regeneration:**Micro‐CT and histology showed significant new bone formation in rHAM‐treated defects, with progressive improvements up to 8 weeks. No bone regeneration observed in PGA‐only controls. – **Periodontal ligament (PDL) regeneration:** Histology and electron microscopy confirmed restoration of PDL with correct fibre orientation and attachment via Sharpey′s fibres in rHAM‐treated sites; controls showed only granulation tissue. – **Cementum regeneration:**rHAM treatment led to deposition of new cementum in correct order; PGA‐only controls showed no cementum formation. – **Overall tissue organisation:**Regenerated tissues (cementum, PDL, alveolar bone) restored normal morphology and organisation, indicating coordinated periodontal regeneration.	
Li et al. (2010)	**Type:** In vitro experimental study **Cells used:** Human periodontal ligament fibroblasts (PDLF), gingival fibroblasts (GF), gingival epithelial cells (GEC) from premolar extraction sites **Study aim:** To investigate the effects of 25kDa recombinant porcine amelogenin (rPAm) on cellular attachment, proliferation, and migration of periodontal and gingival cells **Control:** No rPAm	**Species:** Porcine **Expression system/synthesis method:** GST‐tagged construct in pGEX4T1 expressed in *E. coli* BL21; amelogenin cDNA cloned from pig tooth germ via RT‐PCR; protein induced with IPTG; purified with GSTrap 4B columns **Variants:** 25 kDa recombinant porcine amelogenin (rPAm) **Characterisation:** Purity confirmed by SDS–PAGE, Western blotting, and LC–MS	Likely dissolved in culture media; plates coated with 10 µg/mL rPAm for attachment and proliferation assays	**Cell attachment:** Plates coated with rPAm or collagen; non‐adherent cells counted at multiple early time points (30 min to 4 h) **Cell proliferation:** Cells seeded on rPAm‐coated plates; harvested at 24, 72, and 120 h for counting **Cell migration:** Scratch wound assays; rPAm (10 µg/mL) applied; wound closure monitored every 12 h **Key design/reporting features:** Three human cell types tested; time‐course measurements; multiple in vitro assays employed **Limitations:** Single concentration tested; in vitro only; no comparison with commercial enamel matrix derivatives (Emdogain)	**In vitro biological responses and biomolecular markers:**	The study revealed that the 25‐kDa recombinant amelogenin affects the attachment, proliferation, and migration of the different cell types found in the periodontal unit (PDLF, GF, and GEC).
					– **Cellular Attachment:** Enhanced early attachment of PDLF and GF (30 min); inhibited GEC attachment; no effect at later time points – **Proliferation:** Promoted proliferation of PDLF and GF; suppressed proliferation in GEC – **Migration:** Increased migration in PDLF; reduced migration in GF and GEC at later stages	
Zhang et al. (2010)	**Type:** In vitro experimental study **Cells used:** Murine dental follicle cells (from developing molars of CD‐1 mice) and cementoblasts (OCCM‐30) **Study aim:** To determine whether LAMP‐1 serves as a cell surface binding site for full‐length recombinant mouse amelogenin on tooth root/periodontium‐associated mesenchymal cells **Control:** Emdogain (EMD) and His‐tagged control protein (Positope, Invitrogen)	**Species:** Mouse **Expression system/synthesis:** Not described; rp(H)M180 (180 aa) with 6×His tag provided by Dr. Malcolm Snead **Variants/tag:** Full‐length 180 aa amelogenin with 6×His tag **Characterisation:** His tag confirmed not to alter bioactivity; control protein used to assess non‐specific binding	Direct application: rp(H)M180 dissolved in culture media	**Dose–response binding assay:** 0–100 µg/mL rp(H)M180, saturation at 50 µg/mL **Time‐course:** 0–120 min at 4°C to determine peak binding **Competitive binding:** 20‐fold excess EMD to test specificity **Co‐localisation:** rp(H)M180 and LAMP‐1 visualised via confocal microscopy; pixel overlap quantified **Controls:** His‐tagged protein and EMD included **Limitation:** In vitro only; no in vivo validation	**In vitro biological responses and biomolecular markers:**	The study suggests that LAMP‐1 can serve as a cell surface binding site for recombinant amelogenin and EMD on dental follicle cells and cementoblasts, where these cells are involved in periodontal regeneration.
					– Dose‐dependent binding of rp(H)M180 to cell surface of dental follicle cells and OCCM‐30 cementoblasts – Competitive inhibition with EMD (79% reduction) indicated shared receptor – Confocal microscopy confirmed co‐localisation of rp(H)M180 with LAMP‐1, suggesting receptor‐mediated interaction	
Jingchao et al. (2011)	**Type:** In vitro experimental study **Cells used:** Human bone marrow‐derived stromal cells (hBMSCs) **Study aim:** To investigate the effects of human amelogenin transduction on the regulation of genes involved in osteogenic differentiation **Control:** hBMSCs transduced with lentiviral GFP (LV‐GFP)	**Species:** Human **Expression system/synthesis:** Lentiviral transduction; human amelogenin X cDNA subcloned into FUGW transfer vector to generate FUAmW plasmid **Variants/tag:** Not specifically tagged; EGFP reporter encoded by lentiviral vector **Characterisation:** Transgene expression confirmed by RT‐PCR and flow cytometry; virus titre 5 × 10⁷–1 × 10⁸ IU/mL	Directly dissolved in culture media	– hBMSCs transduced at passage 3 with LV‐hAm or LV‐GFP – Cells selected with 800 µg/mL Zeocin – Transgene expression confirmed after 72 h via flow cytometry and RT‐PCR – Whole‐genome expression profiling using Illumina BeadArray – Differential gene expression filtered for osteogenic relevance – Four genes (BMP‐2, BMP‐6, OPN, VEGFC) validated by qRT‐PCR in triplicate	**In vitro biological responses and biomolecular markers:**	This study revealed that recombinant amelogenin may have a role in regulating hBMSCs osteogenic differentiation as BMP‐ 2, BMP‐6, OPN and VEGFC were up‐regulated.
					– Upregulation of osteogenesis‐related genes BMP‐2, BMP‐6, OPN, VEGFC in hBMSCs after LV‐hAm transduction – Genome‐wide expression profiling used to identify differentially expressed genes – qRT‐PCR confirmed osteogenic gene upregulation	
				**Limitation**: In vitro study; findings may not fully represent in vivo cellular interactions or tissue regeneration		
Kémoun et al. (2011)	**Type:** In vitro experimental study **Cells used:** Human periodontal ligament (hPDL) cells isolated from extracted premolars **Study aim:** To investigate the effects of enamel matrix derivative (EMD), recombinant amelogenin, and BMP2/7 on hPDL cell proliferation, migration, differentiation, and expression of CD146, CD106 and MSCA‐1 **Control:** Recombinant mouse amelogenin (rp(H)M180) and BMP2/7	**Species:** Mouse (rp(H)M180) **Expression system/synthesis:** Not described **Variants/tag:** Full‐length amelogenin; details of post‐translational modifications or tags not reported **Characterisation:** Not reported	Directly dissolved in culture media	– Cell proliferation measured via doubling time and DNA quantification (Qbit dsDNA) over 6 days – Cell cycle analysed by Hoechst staining and FACS – In Vitro wound healing assessed by scratch assay; nuclei counted with ImageJ – Differentiation assays performed in osteogenic, chondrogenic, and adipogenic media – Osteogenic differentiation assessed via immunofluorescence, dot blot, FACS, alkaline phosphatase activity, and mineralisation assays	**In vitro biological responses and biomolecular markers:**	This study demonstrates that CD146(+) hPDL cells are highly responsive to regenerative stimuli and key to proliferation, migration, and differentiation in vitro. BMP‐related pathways (especially when modulated with rp(H)M180 and EMD) drive these effects, making CD146 a potential marker for selecting potent periodontal progenitor cells. Additionally, the study reveals that differentiation toward osteo/cementogenic lineages involves transient changes in the CD146(+) CD106(+) and CD146(+) MSCA‐1(+) subpopulations.
					– **Proliferation:** rp(H)M180, EMD (± rhNoggin), rp(H)M180+rhBMP2/7, and growth medium increased DNA content; BMP2/7 alone, dexamethasone, and low serum had minimal effect – **Cell cycle:** Interventions (except rhBMP2/7) increased S and G2/M phase cells – **Migration/wound healing:** EMD ± rhNoggin, rp(H)M180+rhBMP2/7, and GM accelerated wound closure; effects correlated with CD146(+) cell fraction – **Differentiation:** Osteogenic/cementogenic markers (AP activity, calcium deposition) enhanced by EMD and rp(H)M180+rhBMP2/7; rp(H)M180 alone showed weak but significant effect; rhNoggin partially inhibited EMD‐induced differentiation, implicating BMP signalling – **Marker expression:** Transient changes in CD146(+), CD106(+), and MSCA‐1(+) subpopulations were observed	
				**Limitation**: In vitro study; isolated PDL cells may contain mixed subpopulations, potentially affecting variability		
Cheng et al. (2012)	**Type:** In vitro experimental study **Cells used:** Human periodontal ligament fibroblasts (PDLFs) isolated from extracted premolars **Study aim:** To compare the biological cellular responses of human PDLFs to recombinant full‐length human amelogenin (rHhAm175) versus porcine EMD (pEMD) **Control:** Porcine EMD (pEMD)	**Species:** Human **Expression system/synthesis:** *P. pastoris* yeast expression system; human amelogenin X cDNA encoding the 175‐aa mature protein with N‐terminal His tag subcloned into pPIC3.5K, transformed into *P. pastoris* GS115, induced by methanol **Variants/tag:** N‐terminal hexa‐histidine tag with PreScission protease cleavage site **Characterisation:** SDS‐PAGE, Coomassie staining, Western blot, and mass spectrometry	Directly dissolved in culture media	**Cell attachment:** PDLFs seeded on rHhAm175 (10 µg/mL) or pEMD (200 µg/mL) coated plates, assessed at 30, 60, 120, 240 min **Migration:** Wound healing assay monitored every 12 h for 48 h **Proliferation:** MTT assay over 8 days; BrdU incorporation for DNA synthesis with or without ERK inhibitor PD98059 **Signalling**: Western blot for ERK1/2, phospho‐ERK1/2, Akt, phospho‐Akt **Limitations**: In vitro only; variability in isolated PDL cells from extracted teeth	**In vitro biological responses and biomolecular markers:**	This study revealed that rHhAm175 modulated cell activities of human PDLFs, to a comparable extent as porcine EMD. These data suggest that rHhAm175 might be used to induce periodontal tissue regeneration.
					– **Cell attachment:** rHhAm175 and pEMD enhanced attachment within the first 60 min; ~80% attachment by 2 h – **Cell migration:** Both proteins increased wound closure > 80% at 24 h versus < 25% in control – **Cell proliferation and DNA synthesis:** Dose‐dependent increase with rHhAm175 and pEMD; BrdU incorporation blocked by ERK inhibitor – **Signalling:** ERK1/2 dual phosphorylation induced by rHhAm175 and pEMD; Akt activation not observed	
Tanimoto, Kunimatsu et al. (2012) and Tanimoto, Huang et al. (2012)	**Type:** In vitro experimental study **Cells used:** Human bone marrow‐derived mesenchymal stem cells (MSCs) **Study aim:** To evaluate the effect of recombinant human full‐length amelogenin (rh174) on osteogenic differentiation, including bone marker expression, ALP activity, calcium deposition, and mineralisation **Control:** Commercially available EMD (Emdogain) or no rh174	**Species:** Human **Expression system/synthesis:** *E. coli* BL21; amelogenin cDNA (exons 1–7) subcloned into pRSET A; expression induced with IPTG (0.8 mM) at 37°C for 4 h; purified from inclusion bodies **Characterisation:** SDS‐PAGE, Western blot, MALDI‐TOF mass spectrometry **Variants/tag:** Full‐length 174‐aa protein	Directly dissolved in culture media	**Gene expression:** qRT‐PCR for ALP, OPN, COL1, BSP, OCN normalised to GAPDH **ALP activity:** Measured with pNPP Phosphatase Assay Kit at 405 nm **Calcium deposition:** Measured at 570 nm after HCl dissolution **Mineralisation:** Alizarin Red S staining quantified at 405 nm; dose‐response assessed (0, 10, 100 ng/mL rh174) **Limitations:** In Vitro only; Emdogain comparison limited to mineralisation assay	**In vitro biological responses and biomolecular markers:**	The study reveals that rh174 enhances the mineralisation accompanied by the upregulation of bone markers in human bone marrow MSCs during osteogenic differentiation suggesting the role of amelogenin in regeneration.
					– **Cell differentiation:** rh174 upregulated bone markers (ALP, COL1, OPN, BSP) during osteogenic differentiation; OCN unchanged – **ALP activity:** Significantly higher in rh174‐treated MSCs (days 14–26) – **Calcium deposition:** Elevated in rh174‐treated MSCs from day 18 onward – **Mineralisation:** Dose‐dependent enhancement with rh174; strongest with 100 ng/mL; EMD also enhanced mineralisation	
Tanimoto, Huang et al. (2012) and Tanimoto, Kunimatsu et al. (2012)	**Type:** In vitro experimental study **Cells used:** Human cementoblasts (HCEMs; immortalised with hTERT) and human periodontal ligament cells (HPDLs; isolated from extracted premolars) **Study aim:** To evaluate the effect of recombinant human full‐length amelogenin (rh174) on mineralisation, including gene and protein expression, ALP activity, calcium deposition, and overall mineralisation **Control:** No rh174	**Species:** Human **Expression system/synthesis:** *E. coli* BL21; amelogenin cDNA (exons 1–7) subcloned into pRSET A; expression induced with IPTG (0.8 mM) at 37°C for 4 h; purified from inclusion bodies **Characterisation:** SDS‐PAGE, Western blot, MALDI‐TOF mass spectrometry **Variants/tag:** Full‐length 174‐aa protein	Directly dissolved in culture media	**Gene expression:** qRT‐PCR for ALP, OCN, BSP, normalised to GAPDH; 12‐h treatment **Protein expression:** Western blot for OCN and BSP **ALP activity:** pNPP assay, absorbance at 405 nm **Calcium concentration:** Measured at 570 nm after HCl dissolution **Mineralisation:** Red dye staining after fixation; dose‐response assessment **Limitations:** In vitro only; variability in isolated HPDL populations; no comparison with Emdogain	**In vitro biological responses and biomolecular markers:**	The study demonstrated that rh174 enhances mineralisation and upregulates mineralisation markers in HCEMs, but has no effect on mineralisation in HPDLs, indicating that amelogenin may exert different effects on periodontal ligament (PDL) and cementum.
					– HCEMs: rh174 upregulated ALP, OCN, BSP mRNA and protein; increased ALP activity, calcium deposition, and mineralisation in a dose‐dependent manner – HPDLs: rh174 had no significant effect on gene/protein expression, ALP activity, calcium deposition, or mineralisation	
Lee et al. (2014)	**Type:** Combined in vitro and in vivo experimental study **Cells used:** Human dental pulp stem cells (DPSCs), periodontal ligament stem cells (PDLSCs), and alveolar bone stem/progenitor cells (ABSCs) isolated from patients **Animal model:** 10‐week‐old immunodeficient Harlan mice, subcutaneous implantation **Study aim:** To develop a multiphase 3D‐printed scaffold for regenerating dentin/cementum, PDL, and alveolar bone with spatiotemporal delivery of recombinant human amelogenin, CTGF, and BMP2 **Control:** Empty PLGA microsphere scaffolds (no proteins)	**Species:** Human **Expression system/synthesis:** Not reported **Variants/tag:** Full‐length recombinant human amelogenin **Characterisation:** Not described	**Scaffold:** 3D‐printed PCL/HA (90:10) multiphase scaffolds (5 × 5 × 3 mm) with 100 µm strands and microchannels **Three phases:**	**Cell seeding:** Passage 2–4 cells in collagen solution seeded into scaffolds, 1 h incubation, cultured 2 days *In Vitro* or 4 weeks in defined media **Gene expression:** Real‐time PCR for COL‐I, CEMP1, DSPP, BSP; GAPDH as housekeeping gene **Histology & immunohistochemistry:** H&E, Masson′s Trichrome, Alizarin Red; immunofluorescence for COL‐I, DSPP, CEMP1, BSP; confocal microscopy for 3D tissue analysis **In vivo implantation:** Subcutaneous in immunodeficient mice; retrieval after 6 weeks **Limitations:** Ectopic implantation does not fully replicate clinical periodontal environment; patient‐derived cells may introduce variability due to mixed populations	***In Vitro*** **biological responses and biomolecular markers:**	This study shows that multiphase scaffolds with region‐specific microstructures and spatiotemporal delivery of bioactive cues can effectively regenerate complex periodontal tissues, including dentin/cementum, periodontal ligament, and alveolar bone. The study highlights that the incorporation of specific bioactive proteins (recombinant amelogenin, CTGF, BMP2) can result in coordinated tissue regeneration both in vitro and in vivo.
					– Scaffold‐supported DPSCs, PDLSCs, and ABSCs generated tissue‐specific mineralisation in respective scaffold phases – Enhanced expression of COL‐I (Phase B), DSPP/CEMP1 (Phase A), and BSP (Phase C) in response to bioactive protein delivery	
			1. Phase A (cementum/dentin, 100 µm) – amelogenin 2. Phase B (PDL, 600 µm) – CTGF 3. Phase C (alveolar bone, 300 µm) – BMP2			
					**In Vivo tissue regeneration outcomes:**	
			**Encapsulation:** Bioactive proteins encapsulated in PLGA microspheres and incorporated into each scaffold phase **Sterilisation** with ethylene oxide; visualised with SEM			
					– Integrated tissue formation in subcutaneous implants with distinct dentin/cementum (Phase A, DSPP), PDL‐like (Phase B, CEMP1), and bone‐like (Phase C, BSP) tissues – Empty microsphere scaffolds showed weaker mineralisation and tissue formation	
Olivares‐Navarrete et al. (2014)	**Type:** In vitro experimental study **Cells used:** Human bone marrow‐derived mesenchymal stem cells (MSCs) **Study aim:** To investigate the effects of the N‐terminal amelogenin peptide (NTAP) on osteogenic differentiation, gene expression, and protein secretion in MSCs, compared with recombinant human amelogenin (rAmel) as a control **Control:** Recombinant human amelogenin (rAmel) and vehicle (0.01% acetic acid)	**Species:** Human **Expression system/synthesis:** Commercially sourced from Straumann (Basel, Switzerland) **Variants/tag:** Full‐length recombinant amelogenin (rAmel) **Characterisation:** Not described	Directly dissolved in culture media	**Osteoblastic differentiation:** MSCs treated with 1–10 μg/mL rAmel or NTAP for 24 h; conditioned media collected for ELISA assays of osteocalcin, OPG, TGF‐β1, VEGF **Cell lysates:** DNA quantified (PicoGreen); ALP activity measured and normalised to total protein **Gene expression:** RNA extracted after 12 h, reverse‐transcribed, and analysed via qPCR; normalised to GAPDH **Signal transduction:** Protein phosphorylation assessed by kinase profiler array; PKC, ERK1/2, and β‐catenin activity measured using commercial assays **Inhibitor study:** ERK1/2 inhibition using U0126 to assess pathway involvement in gene expression changes **Limitations:** In vitro findings may not fully represent in vivo interactions; only a single cell type used; effects on other periodontal cell types not assessed; no comparison with commercially available enamel matrix derivatives (Emdogain)	**In vitro biological responses and biomolecular markers:**	This study reveals that NTAP can promote osteogenic differentiation of human MSCs more effectively that full‐length recombinant amelogenin. The implication of this study is that NTAP may be a key active component in EMD driven regeneration.
					– **Cell viability and proliferation:** Neither rAmel nor NTAP affected MSC DNA content – **Early osteogenic markers:** Both increased ALP activity, NTAP more strongly than rAmel – Late osteogenic markers: NTAP induced dose‐dependent increases in osteocalcin, COL1, RUNX2; rAmel had smaller effects – **Secreted factors:** NTAP increased OPG (~75% over control), VEGF (~50%), and TGF‐β1 (modest effect at high dose); rAmel effects were smaller or dose‐dependent – **Signal transduction:** NTAP induced stronger phosphorylation of PKC, ERK1/2, and β‐catenin compared with rAmel; ERK1/2 inhibition blocked NTAP/rAmel‐induced gene upregulation	
Yadegari et al. (2015)	**Type:** In vitro experimental study **Cells used:** L929 mouse fibroblasts; osteoclasts derived from mouse bone marrow **Study aim:** To express and evaluate the biological activity of full‐length recombinant human amelogenin (rhAm) produced in Iranian lizard Leishmania (I.L.L.) as a novel eukaryotic expression system, and compare its effects with commercially available EMD (Emdogain) **Control:** EMD (Emdogain) and no protein	**Species:** Human **Expression system/synthesis:** Novel eukaryotic expression system using Iranian lizard Leishmania (I.L.L.); gene subcloned into pLEXSY‐hyg2 vector with 6His tag; transfected into I.L.L. cells and selected with hygromycin **Variants/tag:** Full‐length human amelogenin with 6His tag **Characterisation:** Confirmed by RT‐PCR, SDS‐PAGE, Western blot; secreted protein purified via Ni‐NTA His‐Bind resin and concentrated	Directly dissolved in culture media	**Osteoclast differentiation:** Bone marrow‐derived osteoclasts cultured in α‐MEM with 10% FBS and 1,25(OH)₂D₃ for 6 days; TRAP+ cells counted under microscope **Proliferation assay:** L929 fibroblasts treated with rhAm (1–10 µg/mL) or EMD; MTT assay used to quantify viability after 4 days **DNA synthesis:** BrdU incorporation measured in L929 cells after 2‐h incubation **Limitations:** In vitro findings may not fully represent in vivo tissue regeneration; single cell type used for proliferation and DNA synthesis; osteogenic effects on other relevant cell types not assessed	**In vitro biological responses and biomolecular markers:**	This study shows that Iranian lizard Leishmania is a viable eukaryotic system for producing recombinant human amelogenin, with advantages like easy handling, low‐cost media, and mammalian‐like modifications. rhAm inhibits osteoclast differentiation and promotes fibroblast proliferation, with more significant effects than EMD on fibroblast proliferation and DNA synthesis.
					– **Cell proliferation:** L929 fibroblasts showed dose‐dependent proliferation with rhAm; EMD promoted proliferation at higher dose – **DNA synthesis:** BrdU incorporation increased with rhAm and EMD; rhAm at 10 µg/mL showed higher effect than EMD – **Osteoclast differentiation:** rhAm and EMD inhibited osteoclast differentiation; TRAP+ mononuclear cells increased with rhAm	
Yoshimi et al. (2016)	**Type:** In vitro experimental study **Cells used:** Human cementoblast‐like cell line (HCEM) **Study aim:** To identify the functional domain of amelogenin responsible for promoting cementoblast proliferation by evaluating various amelogenin cleavage products **Control:** No treatment	**Species:** Human **Expression system/synthesis:** *E. coli* BL21; cDNA subcloned into pRSET A; expression induced with IPTG (0.8 mM, 37°C, 4 h) **Variants:** rh174 (full‐length), rh128 (TRAP domain removed), rh163 (C‐terminal removed), C11 peptide (C‐terminal fragment, commercially synthesised) **Characterisation:** SDS‐PAGE and protein staining	Directly dissolved in culture media	**Cell proliferation assays:** MTS assay (6 days, absorbance at 490 nm) and BrdU assay (24 h, absorbance at 370 nm) **Signalling pathway analysis:** MEK1/2 inhibitor U0126 used to assess ERK1/2 involvement; Western blot for phosphorylated and total ERK1/2 at 30 min post‐treatment **Limitations:** In vitro findings may not fully represent in vivo tissue regeneration; only single cell type used; no comparison with commercially available EMD	**In vitro biological responses and biomolecular markers:**	The study demonstrates that C‐terminal amelogenin peptides (rh128 and C11) significantly enhance proliferation of human cementoblast lineage cells (HCEM) via the MAPK–ERK pathway. This suggests the C‐terminal domain plays a critical role in periodontal tissue regeneration and may be a promising therapeutic target.
					– **Cell proliferation:** rh174, rh128, and C11 significantly increased HCEM proliferation in a dose‐dependent manner; rh163 had no effect – **Signal transduction:** ERK1/2 phosphorylation increased with rh128 and C11; U0126 blocked proliferation induced by active peptides	
Kunimatsu et al. (2017)	**Type:** In vitro experimental study **Cells used:** Human cementoblast‐like cell line (HCEM) **Study aim:** To identify the active site region of amelogenin responsible for promoting osteogenic differentiation and mineralisation **Control:** No treatment	**Species:** Human **Expression system/synthesis:** *E. coli* BL21; cDNA subcloned into pRSET A; expression induced with IPTG (0.8 mM, 37°C, 4 h) **Variants:** rh128 (TRAP domain removed), rh163 (C‐terminal removed), C11 peptide (C‐terminal fragment, commercially synthesised) **Characterisation:** SDS‐PAGE and protein staining	Directly dissolved in culture media	**Osteogenic induction:** α‐MEM with 10 nM dexamethasone, 10 mM β‐glycerophosphate, 50 µg/mL ascorbic acid **Gene expression:** qRT‐PCR for ALP, OCN, BSP; normalised to GAPDH **ALP activity:** Colorimetric assay at 405 nm after 14 days **Calcium quantification:** Colorimetric assay at 610 nm after 21 days **Mineralisation assessment:** Alizarin Red S staining after 21 days **Limitations:** In vitro findings may not fully represent in vivo tissue regeneration; only single cell type used; no comparison with full‐length rh174 or commercial EMD	**In vitro biological responses and biomolecular markers:**	The study reveals that the C‐terminal region of amelogenin, specifically rh128 and the C11 peptide, significantly enhances osteogenic differentiation and mineralisation in human cementoblast lineage cells (HCEM), highlighting its potential application in periodontal tissue regeneration.
					– **Gene expression:** rh128 and C11 significantly upregulated ALP, OCN, BSP mRNA; rh163 had no effect – **ALP activity:** Increased with rh128 and C11, unchanged with rh163 – **Calcium deposition:** Enhanced by rh128 and C11, not by rh163 – **Mineralisation:** Increased with rh128 and C11 (Alizarin Red S staining), unchanged with rh163	
Wyganowska‐Swiatkowska, Urbaniak, Lipinski, Szalata and Kotwicka et al. (2017) and Wyganowska‐Swiatkowska, Urbaniak, Lipinski et al. (2017)	**Type:** In vitro experimental study **Cells used:** Human tongue squamous cell carcinoma cell line (SCC‐25) **Study aim:** To compare the effects of commercial EMD, porcine recombinant amelogenin (prAMEL), and tyrosine‐rich amelogenin peptide (TRAP) on epithelial cell adherence, proliferation, and migration **Control:** Commercial EMD and no treatment	**Species:** Porcine (prAMEL), TRAP derived from porcine AMEL sequence **Expression system/synthesis:**	Directly dissolved in culture media	**Cell culture:** DMEM/F12 + 10% FBS, 100 µg/mL penicillin, 400 ng/mL hydrocortisone, 37°C, 5% CO₂ **Adherence and proliferation:** xCELLigence system; E‐Plate 96 for 14 h adherence, proliferation monitored over 48–77 h at multiple time points **Migration:** xCELLigence CIM‐Plate 16 insert over 49 h **Limitations:** In vitro findings may not fully represent in vivo tissue regeneration; single cell type used; effects on relevant periodontal cells not assessed	**In vitro biological responses and biomolecular markers:**	This study revealed that enamel matrix derivatives and its components (prAMEL and TRAP) did not affect the viability, adhesion, proliferation, or migration of SCC‐25 tongue carcinoma cells. EMD showed no significant impact, while prAMEL inhibited cell proliferation and migration in a dose‐dependent manner. TRAP slightly decreased proliferation but did not significantly affect adhesion or migration.
					– **Cell morphology:** No significant changes; spindle‐shaped cells became slightly circular with increased density – **Cell adherence:** No significant differences among EMD, prAMEL, or TRAP at any dose – **Cell proliferation:** prAMEL reduced proliferation at 12.5–50 µg/mL at 24–48 h; TRAP slightly decreased proliferation; EMD had no significant effect – **Cell migration:** No significant differences across all proteins, doses, and time points	
		– prAMEL: GST‐tagged fusion protein; cloned into pGex4T‐1 vector; expressed in E. coli ArcticExpress; purified via GST affinity chromatography; lyophilised; confirmed by SDS‐PAGE – TRAP: Histidine‐tagged; cloned into pET‐22b(+); expressed in E. coli Rosetta 2 (DE3) pLysS; purified via immobilised metal affinity chromatography; eluted with pH gradient; confirmed by SDS‐PAGE				
		**Characterisation:** SDS‐PAGE confirmed purity; final protein concentrations: prAMEL 1 mg/mL, TRAP ~4.7 mg/mL				
Wyganowska‐Swiatkowska, Urbaniak, Lipinski et al. (2017) and Wyganowska‐Swiatkowska, Urbaniak, Lipinski, Szalata and Kotwicka et al. (2017)	**Type:** In vitro experimental study **Cells used:** Human gingival fibroblast cell line (HGF‐1) **Study aim:** To compare the effects of commercial EMD, porcine recombinant amelogenin (prAMEL), and tyrosine‐rich amelogenin peptide (TRAP) on fibroblast proliferation, migration, and cell cycle progression **Control:** Commercial EMD and no treatment	**Species:** Porcine (prAMEL), TRAP derived from porcine AMEL sequence **Expression system/synthesis:**	Directly dissolved in culture media	**Cell proliferation:** xCELLigence system (RTCA) monitored cell index every 15 min over 72 h; concentrations 12.5, 25, 50 µg/mL **Cell migration:** RTCA CIM‐plates over 72 h **Cell cycle analysis:** Flow cytometry with PI staining after 48 h; G0/G1, S, G2/M phases assessed; apoptosis evaluated **Limitations:** In vitro study may not reflect in vivo tissue interactions; single cell type used; effects on relevant periodontal cells not assessed	**In vitro biological responses and biomolecular markers:**	The study shows that EMD, prAMEL, and TRAP modulate gingival fibroblast behaviour. prAMEL had the strongest effect at 50 µg/mL, enhancing proliferation and migration, while EMD and prAMEL also promoted cell cycle progression (G0/G1 decrease, S/G2/M increase). Effects were ligand‐ and dose‐dependent, with prAMEL and TRAP acting differently from EMD.
					– **Cell proliferation:** EMD significantly increased proliferation at all doses; prAMEL only at 50 µg/mL; prTRAP increased proliferation, not significant at 25 µg/mL – **Cell migration:** prAMEL increased migration at all concentrations; prTRAP at 12.5 µg/mL at 60–72 h; EMD had no significant effect – **Cell cycle progression:** EMD and prAMEL decreased G0/G1 phase, increased S and G2/M phases; prTRAP had no significant effect; no apoptosis observed	
		– prAMEL: GST‐tagged fusion protein; pGex4T‐1 vector; expressed in *E. coli* ArcticExpress; purified by GST affinity chromatography; lyophilised; verified by SDS‐PAGE; final concentration 1 mg/mL – TRAP: His‐tagged; pET‐22b(+); expressed in *E. coli* Rosetta 2 (DE3) pLysS; purified by immobilised metal affinity chromatography; SDS‐PAGE confirmed purity; final concentration ~4.7 mg/mL				
		**Characterisation:** SDS‐PAGE confirmed purity and integrity				
Hakki et al. (2018)	**Type:** In vitro experimental study **Cells used:** Mouse cementoblasts (OCCM‐30) **Study aim:** Evaluate the effects of recombinant human amelogenin (rhAMG) on cementoblast proliferation, mineralisation, and expression of mineralised tissue‐ and cementum‐associated genes **Control:** No treatment	**Species:** Human **Expression system:** *E. coli* BL21 (DE3) **Synthesis method:** PCR‐synthesised codon‐optimised 175‐amino acid gene (excluding signal peptide), cloned into pET11a vector, expressed, purified, and validated via SDS‐PAGE, HPLC, and mass spectrometry **Variants/Characterisation:** Full‐length rhAMG; validated for purity and sequence	Directly dissolved in culture media	**Real‐time proliferation** monitored via xCELLigence RTCA over 144 h ⎕ RNA isolation and qRT‐PCR performed to quantify osteogenic/cementogenic markers (OCN, Runx2, COL I, BSP, OPN, CAP, ALP) **Mineralisation** assessed via von Kossa staining **Immunocytochemistry** for OPN, COL I, LAMP‐1 **Confocal microscopy** for f‐actin and β1 integrin **Limitations**: single cell type; no in vivo validation; no comparison with commercial EMD	**In vitro biological responses and biomolecular markers:**	This study demonstrates that high concentrations of rhAMG promote cementoblast proliferation, osteogenic gene expression, and mineralisation in OCCM‐30 cells, suggesting its potential for periodontal regeneration through LAMP‐1–mediated pathways.
					– **Cellular responses:** Proliferation increased significantly at 100,000 ng/mL; f‐actin intensity increased at highest dose; β1 integrin unchanged – **Gene/protein expression:** Dose‐dependent upregulation of BSP, Runx2, COL I, OPN, OCN, ALP, CAP – **Mineralisation:** Enhanced at all rhAMG concentrations, strongest at 10,000–100,000 ng/mL	
Liao et al. (2020)	**Type**: In vitro and in vivo (mouse) experimental study **Cells used**: Human periodontal ligament cells (hPDLCs) from extracted teeth **Animal model**: 4‐week‐old BALB/c nude mice **Study aim**: Evaluate physicochemical, antibacterial, sustained‐release, osteogenic, and cementogenic properties of rhAm‐loaded mHA/CS scaffold for periodontal regeneration **Control:** No recombinant human amelogenin	**Species:** Human **Expression system:** Prokaryotic expression system **Synthesis method:** Details not fully specified; protein purified before scaffold loading **Variants/Characterisation:** Full‐length rhAm; characterised via loading and release from scaffold	**Scaffold:** Mesoporous hydroxyapatite/chitosan (mHA/CS) **Fabrication:** ‐ **mHA**: CTAB and (NH₄)₂HPO₄ with CaCl₂, stirred, reacted at 100°C, vacuum‐dried, calcined ‐ **mHA/CS**: Chitosan mixed with mHA, crosslinked with glutaraldehyde, vacuum‐dried **Protein loading:** 20 µg/mL rhAm incorporated into mHA, CS, or mHA/CS	**Antibacterial assay:** F. nucleatum and P. gingivalis; OD600 and LIVE/DEAD CLSM imaging **Cytotoxicity:** MTT assay on hPDLCs over 7 days **Controlled release:** rhAm release measured from mHA, CS, and mHA/CS scaffolds **Osteogenic assessment (In vitro):** ALP activity, gene expression (RUNX‐2, DLX‐5, OPN), protein expression via Western blot **In vivo study:** hPDLC‐seeded root slices implanted subcutaneously in mice for 8 weeks; histology assessed cementum‐like tissue formation **Limitations:** No comparison with commercial EMD; ectopic implantation; human tooth implantation in mice may limit clinical translation	**In vitro biological responses and biomolecular markers:**	The study demonstrated that a mesoporous hydroxyapatite/chitosan (mHA/CS) scaffold loaded with recombinant human amelogenin (rhAm) offers promising potential for periodontal regeneration. It inhibits periodontal pathogens, enhances rhAm loading and release, and promotes osteogenic differentiation in human periodontal ligament cells. In vivo, it induced cementum‐like tissue formation, combining antibacterial, osteogenic, and cementogenic properties for improved periodontal tissue regeneration.
					– **Cellular responses:** hPDLC proliferation and viability not affected by scaffold cytotoxicity – **Osteogenic differentiation:** ALP activity and gene/protein expression enhanced by rhAm and mHA/CS‐rhAm, strongest in mHA/CS‐rhAm group – **Antibacterial effect:** mHA/CS scaffold dose‐dependently inhibited *F. nucleatum* and *P. gingivalis* – **Controlled release:** Sustained rhAm release from mHA/CS compared to mHA or CS alone	
					**In vivo tissue regeneration outcomes:**	
					– **Cementogenesis:** Histology showed formation of thin cementum‐like tissue in mHA/CS‐rhAm group; other groups had fibrous or no tissue formation	
Yamato et al. (2021)	**Type**: In vitro experimental study **Cells used**: Human periodontal ligament cells (hPDLCs) **Study aim**: Assess effects of geranyl‐geranyl‐acetone (GGA)‐induced Grp78 overexpression and recombinant murine amelogenin (rM180) on hPDLC proliferation, migration, osteogenesis, and cytokine/angiogenic factor expression **Control:** No rM180, no GGA	**Species:** Mouse **Expression system:** *E. coli* (prokaryotic) **Synthesis method:** Full‐length M180 cDNA cloned into vector, expressed as GST‐rM180 fusion, purified, GST cleaved with PreScission protease, endotoxin removed (< 0.03 EU/10 μg) **Variants/Characterisation:** Full‐length M180, GST fusion confirmed, endotoxin‐free	Directly dissolved in culture media	**Proliferation assay:** WST‐8 at 24, 48, 72 h **Gene expression:** qPCR for osteogenic markers, normalised to GAPDH **Osteogenic differentiation:** Alizarin Red S staining after 21 days **Cell migration:** Scratch assay at 24 and 36 h **Microarray and transfection:** Grp78 overexpression/siRNA; analysed angiogenic and regulatory gene expression **Protein analysis:** Western blot for Grp78, Angptl4, Areg, HIF‐1α, PPARδ, CREB, phospho‐CREB, PKA, phospho‐PKA **secretion:** ELISA for IL‐6, IL‐8, MCP‐1, Areg **Angiogenesis assay:** HUVEC tube formation with hPDLC‐conditioned media **Limitations:** Single cell type; in vitro only; no comparison with commercial EMD	**In vitro biological responses and biomolecular markers:**	The study demonstrates that combining GGA and rM180 amelogenin enhances periodontal ligament cell (hPDLC) migration and angiogenesis. GGA upregulates Grp78, activating HIF‐1α, PPARδ, CREB, and PKA pathways to induce Angptl4 and Areg production, while rM180 further boosts migration and angiogenic cytokine release (IL‐8, MCP‐1, IL‐6), and promotes M2 macrophage polarisation to accelerate wound healing. Though osteogenesis was suppressed, the combination therapy shows synergistic effects.
					– **Cell proliferation:** No significant effect from GGA, rM180, or combination – **Migration:** Enhanced by GGA; further increased with rM180 – **Osteogenesis:** Suppressed by GGA; rM180 alone minimal effect – **Gene/protein expression:** GGA induced Grp78, Angptl4, Areg; combination with rM180 increased IL‐6, IL‐8, MCP‐1 – **Angiogenesis:** GGA+rM180 enhanced HUVEC tube formation via secreted factors	
Chackartchi, Imber et al. (2023) and Chackartchi, Bosshardt et al. (2023)	**Type:** Animal experimental study **Animal model:** 18‐month‐old Sinclair miniature female pigs (55–60 kg) **Study aim:** Assess histological effects of recombinant human amelogenin (rAmelX) on periodontal regeneration in gingival recession defects, comparing CAF + rAmelX versus CAF + placebo **Control:** Carrier only (thermo‐sensitive gel without protein)	**Species**: Human **Expression system**: *E. coli* **Synthesis method**: As per the manufacturer (Prudentix), details not fully reported **Variants/Characterisation**: rAmelX; no additional characterisation reported	**Carrier:** Thermo‐sensitive gel (Poloxamer 407), applied directly to defect site at 0.5 mg/mL rAmelX	**Surgical procedure:** Standardised gingival recession defects; coronally advanced flap (CAF) applied with rAmelX or placebo; flaps sutured with 4–0 nylon **Sample collection:** Animals euthanised at 3 months; defects harvested, fixed, dehydrated, embedded in methylmethacrylate, sectioned, stained with toluidine blue/basic fuchsin **Histologic analysis:** Central‐most sections analysed; landmarks included gingival margin, CEJ, apical notch, new cementum, bone **Histometric analysis:** Measurements of defect height, cementum formation, bone formation, junctional epithelium/sulcus depth **Blinding:** Examiner blinded to treatment **Limitations:** Small sample size; minipigs may not fully replicate human responses; no comparison with commercial EMD (Emdogain)	**In vivo tissue regeneration outcomes:**	This study provides clear histological evidence that recombinant amelogenin (rAmelX), when used with a coronally advanced flap, can enhance regeneration of root cementum and periodontal ligament in recession‐type defects. While no difference in bone regeneration was observed.
					– **Cementum formation:** Significantly greater in CAF + rAmelX (4.38 mm) vs. control (3.48 mm) – **Bone formation:** No significant difference (test: 2.15 mm; control: 2.24 mm) – **Periodontal ligament:** Enhanced regeneration and fibre orientation in test group – **Junctional epithelium/sulcus depth:** Slightly lower in test (2.24 mm) vs. control (2.65 mm), not significant – **Clinical observations:** Uneventful healing, normal behaviour, no adverse reactions	
Chackartchi, Bosshardt et al. (2023) Chackartchi, Imber et al. (2023)	**Type:** Animal experimental study **Animal model:** 18‐month‐old Sinclair miniature female pigs (55–60 kg) **Study aim:** Assess histological effects of recombinant human amelogenin (rAmelX) on periodontal regeneration in two‐wall intrabony defects **Control:** Carrier only (thermo‐sensitive gel without protein)	**Species:** Human **Expression system:** *E. coli* **Synthesis method:** Produced as per the manufacturer (Prudentix) **Variants/Characterisation:** rAmelX; no further characterisation reported	**Carrier:** Thermo‐sensitive gel (Poloxamer 407), applied directly to defect site at 0.5 mg/mL rAmelX	**Surgical procedure:** Bilateral two‐wall intrabony defects prepared at mesial aspects of PM4 and PM2; defects treated with CAF + rAmelX or CAF + placebo; flaps sutured with 5–0 nylon **Sample collection:** Animals euthanised at 3 months; bone blocks fixed in 10% formalin, dehydrated, embedded in methylmethacrylate, sectioned mesial‐distally, stained with toluidine blue/basic fuchsin **Histologic analysis:** Central sections analysed; landmarks included gingival margin, CEJ, apical notch, new cementum, bone **Blinding:** Examiner blinded to treatment **Limitations:** Small sample size; minipigs may not fully replicate human responses; no comparison with commercial EMD (Emdogain)	**In vivo tissue regeneration outcomes:**	This study demonstrated that rAmelX promotes periodontal regeneration in intrabony defects, enhancing root cementum and bone formation compared to the placebo. However, there was no significant difference between the test and control groups.
					– **Defect height:** 6.205 ± 0.389 mm (test) vs. 5.823 ± 1.019 mm (control); no significant difference – **New cementum height:** 4.812 ± 1.167 mm (test) vs. 4.390 ± 1.708 mm (control); no significant difference – **New bone height:** 3.508 ± 1.076 mm (test) vs. 2.965 ± 1.127 mm (control); no significant difference – **Junctional epithelium/sulcus depth:** 2.752 ± 0.648 mm (test) vs. 2.323 ± 0.755 mm (control); no significant difference – **Connective tissue adhesion:** 0.103 ± 0.123 mm (test) vs 0.188 ± 0.133 mm (control); no significant difference – **Histologic observations:** Both groups showed new cementum, bone, and PDL fibre formation; test group displayed more favourable outcomes – **Clinical observations:** Uneventful healing, no adverse reactions	
Frasheri et al. (2023)	**Type:** In vitro experimental study **Cell type:** Immortalised Human Oral Keratinocytes (iHOKs) **Study aim:** Investigate the effects of recombinant human amelogenin on proliferation, migration, and morphology of oral keratinocytes **Control:** Cells cultured without amelogenin	**Species:** Human **Expression system:** ClearColi BL21 *E. coli* **Synthesis method:** Codon‐optimised X‐chromosomal amelogenin (175 aa) cloned into pET‐T7 vector, expressed, purified, dialysed, sterilised, concentrated, stored in 0.05% acetic acid (pH 4) at −80°C **Variants/Characterisation:** Serial dilutions prepared for experimentation (10–10,000 ng/mL)	Directly dissolved in culture media	**Proliferation:** Population doubling (PD) and doubling time (DT) measured at days 7, 14, and 21 using Trypan Blue counting **Metabolic activity:** WST‐1 assays at days 7, 14, 21; medium refreshed every 2 days **Migration:** Scratch assay with imaging at 0 and 24 h post‐scratch **morphology:** Monitored over 21 days **Limitations:** Single cell type; in vitro only; no comparison with commercial EMD	**In vitro biological responses and biomolecular markers:**	This study demonstrates that recombinant amelogenin has a dose‐ and time‐dependent effect on the migration, proliferation, and metabolic activity of keratinocytes. The study also suggests that amelogenin could play a role in promoting periodontal regeneration by modulating cell behaviour, specifically by blocking keratinocyte ingrowth into tissue defects.
					– **Cell proliferation:** High concentrations (≥ 5000 ng/mL) significantly reduced proliferation; lower concentrations (10–1000 ng/mL) had minimal or no effect – **Metabolic activity:** Reduced at ≥ 1000 ng/mL at day 7; 10 ng/mL increased activity by day 21 – **Cell migration:** Dose‐dependent inhibition observed; significant at ≥ 1000 ng/mL – **Cell morphology:** Maintained polygonal shape and homogeneous monolayer in all groups	
Hsia et al. (2024)	**Type:** In vitro and in vivo experimental study **Cells used:** Human periodontal ligament cells (hPDLCs) isolated from extracted teeth **Animal model:** Sixteen 6‐week‐old female SD rats (200–250 g) **Study aim:** Evaluate the effects of recombinant human amelogenin (rhAm) delivered via methacrylated hyaluronic acid (HAMA) hydrogel on hPDLC biological function, osteogenic differentiation, and bone regeneration in a rat calvarial defect model **Controls:** PBS and HAMA hydrogel (carrier only)	**Species:** Human **Expression system/synthesis:** Expressed and purified using Ni‐NTA affinity chromatography; identity confirmed by SDS‐PAGE and Western blot **Variants/Characterisation:** Not further described	**Carrier:** Methacrylated hyaluronic acid (HAMA) hydrogels, 1% and 2% w/v, crosslinked with lithium phenyl‐2,4,6‐trimethylbenzoylphosphinate (LAP) under 405 nm light **Method:** rhAm incorporated into HAMA solution before hydrogel formation	**Cell viability and proliferation:** Assessed with CCK‐8 assay at 24, 48, 72 h; growth curve plotted at days 1, 3, 5, 7, 9 **Osteogenic differentiation:** ALP staining, Alizarin Red staining, and RT‐PCR of Runx2, OCN, COL1 after 14 days **Animal model:** Calvarial defects created in 6‐week‐old female rats; four groups: PBS, rhAm, HAMA, rhAm‐HAMA **Analysis:** Micro‐CT for bone volume/density/trabecular number; histology (HE, Masson) at 4 and 8 weeks **Limitations:** Small sample size, no power calculation; rat model may not fully replicate human periodontal bone defects; no comparison with commercial EMD	**In vitro biological responses and biomolecular markers:**	The study demonstrates that rhAm enhanced hPDLC proliferation and osteogenic differentiation in vitro, with significant upregulation of osteogenesis‐related genes, where HAMA hydrogel served as an effective carrier for rhAm, stabilising bone particles and enabling controlled release. In a rat calvarial bone defect model, the rhAm‐HAMA hydrogel group demonstrated superior bone repair, with higher mineralised tissue volume, trabecular number, and mineralised tissue density compared to other groups.
					– **Cell viability/proliferation:** rhAm‐HAMA hydrogel non‐cytotoxic; maintained hPDLC proliferation – **Osteogenic differentiation:** Upregulated ALP activity and expression of Runx2, OCN, COL1 after 14 days	
					**In vivo tissue regeneration outcomes:**	
					– **Bone formation:** rhAm‐HAMA hydrogel enhanced defect repair, with higher mineralised tissue density, trabecular number, and mineralised tissue volume compared to PBS, rhAm alone, and HAMA alone – **Histology:** rhAm‐HAMA group showed uniform, regularly arranged new bone consistent with micro‐CT findings	

### 3.3 Risk of Bias in Studies

Risk of bias was assessed using the QUIN Tool for in vitro studies and SYRCLE's Risk of Bias Tool for animal studies. Results are presented in Tables 2 and 3, respectively. All 20 in vitro studies assessed using the QUIN Tool scored between 50% and 63%, placing them uniformly in the medium risk of bias category. No in vitro study achieved a low‐risk classification (> 70%), and none fell into the high‐risk category (< 50%). This indicates consistent methodological limitations across studies, rather than isolated deficiencies in individual reports. Six animal studies were evaluated using SYRCLE's Risk of Bias Tool. All animal studies were classified as having an overall high risk of bias, as each exhibited at least one domain rated as high risk. High‐risk judgements were most frequently observed in the selection, performance and detection domains. In contrast, attrition bias was commonly rated as low risk, and the ‘other bias’ domain was generally assessed as low or unclear. Reporting bias was variable, with some studies demonstrating adequate outcome reporting and others lacking sufficient detail to permit confident judgement.

**Table 2:** The QUIN Tool for evaluating the risk of bias of in vitro studies.

	Criteria												
Author (year)	1	2	3	4	5	6	7	8	9	10	11	12	Score (%)
Zeichner‐David et al. (2006)	2	0	2	2	2	1	0	2	0	0	1	2	58
Li et al. (2010)	2	0	2	1	2	0	0	2	0	0	2	2	54
Zhang et al. (2010)	2	0	2	2	2	0	0	2	0	0	2	2	58
Jingchao et al. (2011)	2	0	2	2	2	0	0	2	0	0	1	2	54
Kémoun et al. (2011)	2	0	2	2	2	0	0	2	0	0	2	2	58
Cheng et al. (2012)	2	0	2	2	2	0	0	2	0	0	2	2	58
Tanimoto, Huang et al. (2012) and Tanimoto, Kunimatsu et al. (2012)	2	0	2	2	2	0	0	2	0	0	2	2	58
Tanimoto, Huang et al. (2012) and Tanimoto, Kunimatsu et al. (2012)	2	0	2	2	2	0	0	2	0	0	2	2	58
Lee et al. (2014)	2	0	2	2	2	0	0	2	0	0	1	2	54
Olivares‐Navarrete et al. (2014)	2	1	2	2	2	0	0	2	0	0	2	2	63
Yadegari et al. (2015)	1	0	2	2	2	0	1	2	0	0	1	2	54
Yoshimi et al. (2016)	2	0	2	1	2	0	0	2	0	0	1	2	50
Kunimatsu et al. (2017)	2	0	2	1	2	0	0	2	0	0	1	2	50
Wyganowska‐Swiatkowska, Urbaniak, Lipinski et al. (2017) and Wyganowska‐Swiatkowska, Urbaniak, Lipinski, Szalata and Kotwicka (2017)	2	0	2	2	2	0	0	2	0	0	2	2	58
Wyganowska‐Swiatkowska, Urbaniak, Lipinski et al. (2017) and Wyganowska‐Swiatkowska, Urbaniak, Lipinski, Szalata and Kotwicka (2017)	2	0	2	2	2	0	0	2	0	0	2	2	58
Hakki et al. (2018)	2	0	2	1	2	0	0	2	0	0	1	2	50
Liao et al. (2020)	2	1	2	2	2	0	0	2	0	0	2	2	63
Yamato et al. (2021)	1	0	2	2	2	0	1	2	0	0	1	2	54
Frasheri et al. (2023)	2	0	2	2	2	0	0	2	0	0	2	2	58
Hsia et al. (2024)	2	1	2	2	2	0	0	2	0	0	2	2	63

**Table 3:** The SYRCLE′s RoB tool for evaluating the risk of bias of animal studies.

	Domain									
Type of bias	Selection			Performance		Detection		Attrition	Reporting	Other
Author (year)	1	2	3	4	5	6	7	8	9	10
Haze et al. (2009)	N	Y	U	N	N	N	N	Y	N	U
Lee et al. (2014)	N	Y	U	N	U	Y	N	Y	N	Y
Liao et al. (2020)	N	Y	N	N	N	N	N	Y	Y	Y
Chackartchi, Bosshardt et al. (2023) and Chackartchi, Imber et al. (2023)	U	Y	Y	N	U	U	Y	Y	Y	Y
Chackartchi, Bosshardt et al. (2023) and Chackartchi, Imber et al. (2023)	U	Y	Y	N	U	U	Y	Y	Y	Y
Hsia et al. (2024)	N	Y	N	N	N	N	N	Y	U	Y

### 3.4 Evidence Synthesis Across Preclinical Domains

Synthesis was structured around four evidence domains: (1) in vitro biological responses and biomolecular markers, (2) in vivo tissue regeneration outcomes, (3) protein synthesis and characterisation and (4) delivery systems and formulation strategies. These domains were integrated through qualitative narrative mapping, supplemented by a semi‐quantitative effect‐direction (vote‐count) analysis. Owing to substantial methodological heterogeneity, approaches typically applied in systematic reviews of clinical studies were not feasible.

#### 3.4.1 In Vitro Biological Responses and Biomolecular Markers

Across 20 in vitro studies, recombinant amelogenin consistently promoted pro‐regenerative cellular behaviour, particularly in pathways linked to mineralised tissue formation. Enhanced attachment, migration, differentiation, mineralisation and upregulation of osteogenic/cementogenic markers (ALP, osteocalcin, BSP, RUNX2) were frequently reported. The vote‐count synthesis showed the strongest positive signal for osteogenic and mineralisation‐related outcomes, whereas proliferation and migration effects were notably inconsistent.

A wide range of cell types was used, including primary human periodontal ligament cells (fibroblasts, gingival fibroblasts, gingival epithelial cells; 6 studies), human bone marrow stromal cells (3), human cementoblasts (2), murine dental follicle cells (1), mouse PDL cells (1), human dental pulp/PDL/alveolar bone stem‐progenitor cells (1), mouse fibroblasts (1), human carcinoma and gingival fibroblast cell lines (2), mouse cementoblasts (1) and human oral keratinocytes (1). This diversity strengthens biological plausibility but introduces substantial heterogeneity in baseline behaviour, receptor expression and differentiation potential.

Importantly, several studies reported neutral, mixed or dose‐dependent effects, particularly for proliferation and migration, indicating that amelogenin's influence is not uniformly stimulatory and may depend on cell lineage, maturation state or exposure parameters. Variability in assay selection, dosing and time points further limited comparability. Overall, the in vitro evidence supports a reproducible pro‐mineralisation effect, but early‐stage cellular responses remain context‐dependent (Table 4).

**Table 4:** Semi‐quantitative effect‐direction synthesis of recombinant amelogenin outcomes by domain and study type.

Outcome domain	Study type	Positive	No difference/mixed	Negative
In vitro cellular responses	In vitro (*n* = 17)	13	4	0
	Animal (*n* = 3^a^)	2	1	0
Osteogenic/Cementogenic marker upregulation	In vitro (*n* = 17)	11	6	0
	Animal (n = 2^a^)	2	0	0
Mineralisation	In vitro (*n* = 6)	6	0	0
	Animal (*n* = 2^a^)	1	0	0
In vivo periodontal tissue regeneration				
Complete regeneration (bone, cementum, and PDL)	Animal (*n* = 4)	2	2	0
Bone	Animal (*n* = 5)	3	2	0
Cementum	Animal (*n* = 5)	4	1	0
Periodontal ligament	Animal (*n* = 4)	3	1	0

a Animal studies reporting in vitro endpoints in addition to histology.

#### 3.4.2 In Vivo Tissue Regeneration Outcomes

Six animal studies evaluated recombinant amelogenin, collectively indicating a biologically active but incompletely characterised regenerative effect. Cementum formation was the most consistently improved outcome relative to controls, whereas bone formation and PDL organisation showed variable results influenced by defect type, healing duration and delivery method.

Models included miniature pigs (2 studies), beagle dogs (1), Sprague–Dawley rats (1), BALB/c Nude mice (1) and Harlan mice (1), with substantial variation in defect geometry and evaluation protocols. Four studies assessed tri‐tissue regeneration, one focused solely on bone, and one on cementum. This diversity broadens translational relevance but reduces internal consistency and complicates cross‐study comparison.

Histological and histometric analyses dominated, supplemented by radiographic or descriptive assessments. No adverse inflammatory responses were reported, though study designs were not optimised for safety assessment. Vote‐count synthesis indicated consistent positive effects on cementum and PDL regeneration, while complete tri‐tissue regeneration and bone outcomes were mixed (Table 4). Overall, the in vivo evidence supports amelogenin's regenerative potential but does not yet demonstrate predictable, comprehensive periodontal repair.

#### 3.4.3 Protein Synthesis and Characterisation

Protein production and characterisation were major sources of methodological variability. Sixteen studies described synthesis methods, with *E. coli* expression systems used in 15. Recombinant human amelogenin was most common (*n* = 16), followed by murine (*n* = 4) and porcine (*n* = 3) variants. However, few studies examined whether variant‐specific structural differences influenced biological activity.

Characterisation quality ranged from detailed purification and molecular verification to minimal or absent reporting. This inconsistency introduces uncertainty about protein purity, aggregation state, truncation or post‐translational modifications—factors that could meaningfully influence biological outcomes. As a result, heterogeneity in protein quality may partially explain divergent findings across studies.

#### 3.4.4 Delivery Systems and Formulation Strategies

Delivery strategies were evaluated in only a small subset of studies and remain exploratory. Most in vitro work relied on direct dissolution in culture media (*n* = 15), which is useful for mechanistic assays but poorly reflects clinical delivery constraints. Carrier‐based approaches were limited and highly heterogeneous, including Poloxamer 407 hydrogels (2 studies), HAMA hydrogels (1), mHA/CS scaffolds (1), PGA carriers (1) and PCL/HA composites (1). Two studies did not clearly report their delivery method.

These carriers aimed to improve retention, defect adaptation or release kinetics, but the small number of studies and absence of comparative designs prevent any conclusions about optimal delivery. In several cases, it is unclear whether observed benefits were attributable to amelogenin, the carrier or their interaction. Thus, current delivery systems should be viewed as proof‐of‐concept rather than evidence‐based formulation strategies. Table 5 summarises the carrier‐based systems tested in vivo.

**Table 5:** Overview of carrier systems, modes of application, intended carrier functions and reported biological effects of recombinant amelogenin in animal models.

Study	Carrier	Mode of application	Intended role of carrier	Reported biological effects
Haze et al. (2009)	PGA carrier	Injectable; no in situ gelation	Minimally invasive delivery and localised retention	Tri‐tissue regeneration achieved
Lee et al. (2014)	Multiphase 3D‐printed PCL/HA scaffold	Solid implantable matrix	Structural support and sustained spatiotemporal delivery of biological agents	Tissue‐specific regeneration in ectopic model
Liao et al. (2020)	Mesoporous mHA/CS scaffold	Solid implantable matrix	Structural support and sustained local release with antibacterial properties	Enhanced cementum mineralisation
Chackartchi, Bosshardt et al. (2023) and Chackartchi, Imber et al. (2023)	Poloxamer 407	Injectable; in situ gelation (thermosensitive)	Minimally invasive delivery and localised protein retention/release with defect fill/adaptation	Improved retention and handling; modest biological effect
Chackartchi, Bosshardt et al. (2023) and Chackartchi, Imber et al. (2023)	Poloxamer 407	Injectable; in situ gelation (thermosensitive)	Minimally invasive delivery and localised protein retention/release with defect fill/adaptation	Improved retention and handling; modest biological effect
Hsia et al. (2024)	Methacrylated hyaluronic acid (HAMA) hydrogel	Injectable; in situ gelation with photo‐crosslinking	Minimally invasive delivery and localised protein retention/release with defect fill/adaptation	Improved bone/defect fill

### 3.5 Comparative Insights and Methodological Diversity

Across the 23 included studies, a thematic and methodological analysis revealed extensive heterogeneity spanning every domain of investigation, including protein source and characterisation, delivery systems, cell models, defect types and outcome measures. This variability was not incidental but structural, shaping the direction and magnitude of reported effects and reinforcing that recombinant amelogenin (rAmel) functions as a context‐dependent biologic rather than a uniformly stimulatory agent. While many studies reported pro‐regenerative cellular or tissue‐level responses, others demonstrated neutral or even inhibitory effects under specific conditions, highlighting the influence of dose, formulation, cell lineage and model design on observed outcomes. Such diversity in experimental conditions and biological responses underscores the exploratory nature of the current evidence base and necessitates reliance on descriptive synthesis rather than quantitative comparison.

This analysis also identified important gaps in comparative evidence. In vitro studies provided the first detailed but methodologically inconsistent comparisons between rAmel and EMD, with findings suggesting overlapping biological activities but insufficient data to support claims of equivalence or superiority. Notably, none of the animal studies incorporated EMD comparators, limiting the ability to contextualise rAmel's regenerative potential against an established clinical benchmark. Taken together, these patterns position the field as early‐stage and hypothesis‐generating, with current evidence mapping the breadth of investigative approaches rather than defining therapeutic performance.

## 4 Discussion

This scoping review synthesised the preclinical evidence evaluating recombinant amelogenin across in vitro and animal models of periodontal regeneration. Collectively, the included studies demonstrate that rAmel elicits biologically relevant cellular and tissue‐level responses across a range of experimental settings. However, the evidence base remains exploratory, heterogeneous and methodologically limited, precluding definitive conclusions regarding comparative efficacy or clinical effectiveness.

### 4.1 Biological Activity and Regenerative Patterns

Preclinical studies indicate that rAmel consistently modulates key cellular behaviours relevant to periodontal regeneration, including cell attachment, migration, proliferation, differentiation and mineralisation‐related activity in vitro. Early mechanistic work demonstrated that amelogenin can function as a signalling molecule influencing periodontal ligament and cementoblast lineage cells, with effects mediated through receptor‐associated interactions and downstream pathways such as MAPK/ERK and BMP‐related signalling (Cheng et al. 2012; Kémoun et al. 2011; Zeichner‐David et al. 2006; Zhang et al. 2010). Consistent with these observations, multiple studies reported upregulation of genes and proteins associated with osteogenic and cementogenic pathways, supporting the biological plausibility of rAmel in periodontal regeneration (Haze et al. 2009; Taylor et al. 2006). However, the magnitude and consistency of these responses varied substantially between studies, reflecting differences in cell type, protein source, concentration, exposure duration and assay design.

In animal models, rAmel was associated with favourable regenerative outcomes in several studies, particularly with respect to cementum formation and periodontal ligament organisation (Haze et al. 2009; Lee et al. 2014). Bone regeneration outcomes were more variable and appeared dependent on defect configuration, healing duration and delivery strategy. Importantly, not all studies evaluated all components of periodontal regeneration, limiting cross‐study comparability. Effect‐direction synthesis indicated that positive or mixed effects predominated across outcome domains, but neutral findings were also observed, particularly for bone regeneration and complete tri‐tissue repair. These findings suggest that rAmel produces predominantly favourable, but defect‐dependent regenerative responses rather than consistent or predictable efficacy across models.

### 4.2 Comparison With Enamel Matrix Derivative and Other Biologics

Only a few studies directly compared rAmel with EMD, and these comparisons were limited to cell culture models. Nevertheless, rAmel elicited biological responses broadly aligned with those reported for EMD for selected outcomes. For instance, Tanimoto, Huang et al. (2012) and Tanimoto, Kunimatsu et al. (2012) reported remineralisation effects comparable to Emdogain at a 10‐fold lower concentration of rAmel, and Wyganowska‐Swiatkowska, Urbaniak, Lipinski et al. (2017) and Wyganowska‐Swiatkowska, Urbaniak, Lipinski, Szalata and Kotwicka (2017) demonstrated enhanced inhibition of epithelial proliferation and increased fibroblast migration with recombinant porcine amelogenin. However, differences in experimental conditions, protein formulation, and delivery methods limit meaningful equivalence or superiority claims. Other studies suggested that EMD or multi‐component biologics may exert more robust or consistent effects, potentially reflecting the contribution of additional enamel‐derived or signalling components absent in isolated recombinant proteins. Kémoun et al. (2011) showed that EMDs significantly enhanced proliferation, migration, wound healing and differentiation of human periodontal ligament cells, whereas mouse rAmel directly dissolved in media had only a weak effect. Moreover, when recombinant bone morphogenetic proteins (BMPs) were added to rAmel, cellular responses were significantly enhanced, suggesting a role for BMP‐related signalling (Kémoun et al. 2011). In addition to BMPs, other biologics such as recombinant human platelet‐derived growth factor (rh‐PDGF) are supported by high‐quality multicentre clinical trials in periodontal regeneration. This review identified no direct comparisons between rAmel and these agents (Nevins et al. 2013). As such, the relative positioning of rAmel within the broader landscape of regenerative biologics remains undefined.

### 4.3 Delivery Systems as a Critical Determinant

Delivery strategy emerged as a key contextual factor influencing reported outcomes. In contrast to Emdogain, which is standardised with a single carrier (PGA), rAmel has been tested in conjunction with thermosensitive hydrogels (e.g., Poloxamer 407), light‐activated scaffolds (e.g., HAMA) and bioactive scaffold‐based systems (e.g., PCL/HA, mHA/CS). These carriers were primarily explored for feasibility, retention and biological compatibility rather than comparative performance. Several studies employing injectable or crosslinked matrices reported more spatially organised or sustained regenerative responses; however, carrier bioactivity and the absence of appropriate carrier‐only controls complicate interpretation. As a result, it remains difficult to isolate the independent contribution of rAmel from that of the delivery vehicle. For example, the modest clinical gains reported by Chackartchi, Bosshardt et al. (2023) and Chackartchi, Imber (2023) using rAmelX in Poloxamer 407 compared to control may partially reflect carrier‐mediated effects. The available evidence, therefore, supports the interpretation of rAmel delivery systems as exploratory platforms rather than validated formulations.

### 4.4 Additional Methodological Limitations and Sources of Bias

Interpretation of the evidence is further limited by methodological weaknesses that extend beyond the heterogeneity described earlier. Most of the included studies were in vitro, yet many lacked essential reporting needed to assess experimental quality, including protein characterisation, dosing rationale and degradation or release behaviour. Core controls, such as carrier‐only, dose–response or negative controls, were frequently absent, making it difficult to attribute observed effects specifically to rAmel. In vitro studies also commonly omitted details required for reproducibility, such as passage number, culture conditions and assay validation, contributing to consistently medium QUIN scores. The animal studies showed similar issues, with small sample sizes, limited reporting of randomisation or blinding and inconsistent justification for defect models or healing intervals, resulting in high or unclear risk of bias across multiple SYRCLE domains. Collectively, these deficits reduce confidence in causal inference and prevent meaningful comparison across studies, necessitating reliance on narrative and effect‐direction synthesis rather than quantitative analysis.

### 4.5 Implications for Translation and Future Research

Taken together, the available studies suggest that rAmel exhibits biologically meaningful activity in preclinical settings, indicating potential as a regenerative agent. However, the evidence remains insufficient to determine its clinical relevance or to position it relative to established therapies. At this stage, rAmel is best regarded as an emerging candidate whose future development will depend on greater molecular standardisation, clearer characterisation of dose–response and degradation behaviour and delivery systems supported by appropriate controls. Amelogenin‐derived peptides such as the C‐terminal C11 fragment and the tyrosine‐rich amelogenin peptide (TRAP) have demonstrated strong osteogenic and mineralisation effects in human cementoblast lineage cells (Kunimatsu et al. 2017; Yoshimi et al. 2016) and may warrant exploration alongside full‐length rAmel (Fiorino et al. 2021). The recombinant nature of rAmel also offers practical advantages by avoiding reliance on animal‐derived materials. To advance the field, future studies should prioritise rigorous protein characterisation, controlled evaluation of delivery platforms, defect models aligned with periodontal biology and direct comparisons with established biologics, including EMD. Well‐designed animal studies and early‐phase clinical trials will be needed to determine whether the biological signals observed to date translate into reproducible therapeutic benefit.

## 5 Conclusion

The available preclinical evidence indicates that recombinant amelogenin (rAmel) can elicit biologically meaningful responses relevant to periodontal regeneration, including osteogenic and cementogenic activity in vitro and elements of cementum and periodontal ligament formation in selected animal models. These effects, however, were not uniform and varied according to protein source, formulation, dose, delivery strategy and experimental context. Limited and methodologically inconsistent comparisons with EMD did not permit conclusions regarding equivalence or superiority, and most delivery systems were exploratory, making it difficult to disentangle rAmel‐specific effects from those of the carrier.

Taken together, rAmel should be regarded as an emerging regenerative biologic with promising but directional preclinical activity and no established clinical effectiveness. Its translational potential will depend on more rigorous and standardised protein production, clearer definition of dose–response and degradation behaviour, validated delivery platforms with appropriate controls and direct comparisons with established biologics. Well‐designed animal studies and early‐phase clinical trials will be essential to determine whether the encouraging biological signals observed to date can be translated into predictable and clinically meaningful outcomes.

## Author Contributions

Denny Luo contributed to the conceptualisation and methodology of the review, protocol registration, literature search, study selection, data extraction, risk of bias assessment, data synthesis and drafting of the manuscript. Jennifer Mardini contributed to study selection cross‐checking, verification of data extraction and risk of bias assessments and critical revision of the manuscript. Raymond Lee contributed to resolving reviewer discrepancies, providing methodological oversight and critically revising the manuscript. Fariba Dehghani and Aaron Schindeler contributed to conceptual guidance, supervision and critical revision of the manuscript.

## Funding

The authors received no specific funding for this work.

## Conflicts of Interest

The authors declare no conflicts of interest.

## Data Availability

All data generated or analysed during this review are included in this published article and its supplementary materials. No datasets were generated for quantitative meta‐analysis. A semi‐quantitative effect‐direction (vote‐count) synthesis was performed by tabulating outcomes across included studies in spreadsheet format, and no bespoke analytic code was used. Additional materials or clarifications related to the data presented in this review are available from the corresponding author upon reasonable request.
